# Export of microRNAs: A Bridge between Breast Carcinoma and Their Neighboring Cells

**DOI:** 10.3389/fonc.2016.00147

**Published:** 2016-06-20

**Authors:** Devashree Jahagirdar, Shruti Purohit, Aayushi Jain, Nilesh Kumar Sharma

**Affiliations:** ^1^Cancer and Translational Research Lab, Dr. D.Y. Patil Biotechnology & Bioinformatics Institute, Dr. D.Y. Patil Vidyapeeth, Pune, India

**Keywords:** non-coding short RNA, microRNA, breast tumor, signaling, extracellular vesicles, targeting, breast carcinoma

## Abstract

Breast cancer is a leading type of cancer among women in India as well as worldwide. According to the WHO 2015 report, it has been anticipated that there would be a twofold rise in the death due to breast cancer among women. The heterogeneous property of breast carcinoma has been suggested to be linked with dedicated set of communication and signaling pathway with their surroundings, which culminate into progression and development of the cancer. Among the plethora of communication tools in the hand of breast carcinoma cells is the recently appreciated exocytosis of the tightly packed short non-coding RNA molecules, predominantly the microRNAs (miRNAs). Recent studies suggest that miRNAs may work as courier messengers to participate in endocrine and paracrine signaling to facilitate information transfer between breast carcinoma and their neighboring cells. Evidence suggests that breast tumor cells communicate *via* packaged miRNAs in the tumor-released microvesicles, which enrich the tumor microenvironment. There is a strong view that dissecting out the mechanistic and regulatory aspects of miRNA export and role may uncover many prospects for overcoming the signaling defects and thereby controlling aberrant cell division. The detection of circulating miRNAs associated with breast carcinoma can also be used as biomarkers for early diagnosis. This review article is an attempt to provide updated knowledge on implications of short RNAs and their transport in the breast cancer pathophysiology.

## Introduction

According to the WHO World Cancer Report 2015, there are about 14 million newly diagnosed and 8.2 million cancer-related mortality cases worldwide (1). Among several types of cancer, breast cancer is the most noticeable carcinoma among women in developed and developing countries, where estimated cases until 2012 were 1.7 million that resulted in 521,900 deaths, accounting for 25% of all cancer cases and 15% death in females (1–3). The bottleneck to surmount breast cancer lies in drugs/inhibitors resistance, aberrant signaling, appetite to continuously grow, and manipulated communication skills among the breast carcinoma cells (4, 5).

Among several signaling approaches, one is autocrine where the signal is produced and accepted by the same cell. In case of juxtacrine signaling, the signal is only received by the adjacent cells through the cell membrane *via* membranous lipids and integral proteins. In addition to autocrine and juxtacrine, paracrine signaling involves signal transfer to the number of cells in its vicinity and endocrine signaling deals with the target cells placed at a distance involving circulation through blood (6, 7). Several types of cancer cells, including breast carcinoma, communicate with each other as well as their neighboring cells *via* number of signaling pathways, including NF-κB (7, 8), Hedgehog (9), phosphatidylinositol 3-kinase-Akt (PI3K-Akt) (7, 10), Notch-epidermal growth factor receptor (Notch-EGFR) (11), and short non-coding RNA export and import (12–14). The tumor microenvironment has a crucial part in the survival and growth of the cancer cells (15, 16).

The success or failure of these pathways is a key to regulate cell growth, proliferation, differentiation, apoptosis, and resistance against genotoxic insults (17). Small non-coding RNAs are a group of highly conserved molecules responsible for controlling gene expression and dictating cellular signaling. microRNAs (miRNAs) as a type of short non-coding RNAs with a sequence of 21–25 nt have widely been appreciated for their role in transcriptional repression, mRNA degradation, and extra-environmental signaling (13, 18, 19). Recently, there are sufficient data pointing to upregulation and downregulation of miRNAs in setting up the platform for augmented pathophysiology of breast carcinoma (20). The concerted efforts of protein family cause the miRNA to be stabilized in the exterior region of the cells, making it accessible to get transported to local vicinity (13, 14, 19). Currently, signaling pathways mediated by miRNAs have garnered wide attention in breast cancer pathophysiology. The abnormal exchange of signals involving non-coding short RNAs, including miRNAs, are found to create tumor-friendly microenvironment (21). One of the approaches to establish bridges between breast carcinoma and their microenvironment cellular community is exocytosis of tightly packed miRNAs (22–25). The current understanding and consensus have pointed out that distribution and expression of miRNAs are of utmost importance for disease prognosis and potential target in cancer therapy (26, 27).

In this review, we summarize biogenesis of miRNAs and their export mediated by exosome to target the neighboring cells in the microenvironment. The focus of this paper is to mark the miRNAs as a potent extracellular signaling molecule responsible for breast carcinoma pathophysiology.

## Cell Signaling Overview in Breast Tumor

Signaling is the key element for any cell to survive, grow, and proliferate. The mechanism of signaling is categorized into four types, autocrine, paracrine, juxtacrine, and endocrine signaling, which is described in the Section “Introduction.” Signaling pathways are based on the ligand–receptor binding complex, which leads to a much stronger downstream signal, thereby regulating the genetic expression. Signaling upon binding of a ligand in the cells mostly involves phosphorylation of the receptor at specific amino residues, which recruits various intracellular proteins amplifying the intensity toward the nucleus. There are a number of signaling pathways that are responsible for the cells growth, differentiation, proliferation, and also for resisting apoptosis and further metastasizing (28).

One of the signaling pathways is Notch pathway that is responsible for survival, growth, and proper development of cells. Notch is a transmembrane receptor, which has a strong affinity for the delta ligands and with its proteolytic cleavage travels to the nucleus and acts as transcriptional regulator activating gene expression. Some miRNAs either act as the notch receptor inhibitors or as the nuclear transcriptional inhibitors. As such, notch receptor inhibitor is miRNA-141, whereas Snail and zinc finger E-box binding homeobox 1 (ZEB1) transcriptional factors are inhibited by miRNA-34a (11). miRNA-374a is found to be overexpressed in Wnt/β-catenin pathway. For example, miRNA-451 acts as a potent inhibitor for the downstreaming signaling of the Wnt/β-catenin, decreasing the levels of β-catenin (29). Hedgehog is a developmental signaling pathway, which focuses on the embryogenesis, carcinogenesis, and tissue repair caused due to chronic inflammation. The oncogenic miRNA cluster family (miRNA17–92) regulates the Hedgehog signaling (9). Signaling pathway NF-κB plays a crucial role in the cell proliferation, tumor development, inflammation, and also regulation of the innate and adaptive immune response (8). The pathway is also known to be guided by the expression of different miRNAs. There are several mechanisms by which NF-κB pathway is regulated. miRNA-21 targets signal transducer and activator of transcription 3 (STAT3), which is downregulated. Furthermore, miRNA-21 targets phosphatase and tensin homolog (PTEN), thereby inhibiting Akt phosphorylation and promoting NF-κB activation (30).

Cancer-associated fibroblasts are the stromal cells, non-malignant in nature, which arise from the differentiation of the progenitor cells to promote tumor health by satisfying their nutrient needs (31, 32). Highly activated growth and proliferation of the cancer cells is due to unregulated signaling pathways. The tumor microenvironment helps the cancer cells for this aberrant signaling, as these cells are highly dependent on the microenvironment for their increased need of energy for their proliferation. This tumor microenvironment is solely responsible for causing apoptosis and drug resistance by cell–cell communication *via* some matrix soluble factors enhancing the cellular growth and its development (33). Overexpression of soluble growth factor such as cytokines, mediated by cancer-associated fibroblast cells, acting as nutrient benefactor for the cells can decrease the integrin expression, further leading to metastatic progression (33).

## Types of Non-Coding RNAs

**Figure 1:**
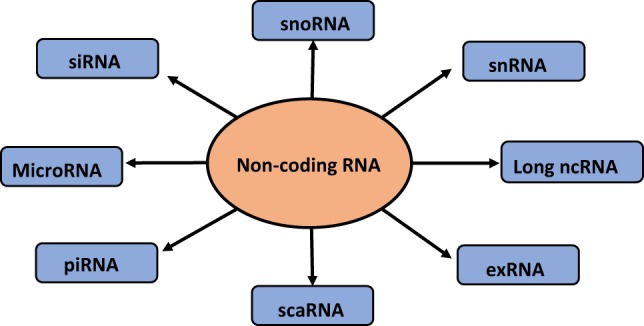
**Types of non-coding RNAs**.

Short non-coding RNAs consist of 1% of the genome (34), with size ranging from 20 to 25 nt. Small non-coding RNAs consist of miRNA, small interfering RNA (siRNA), piwi-interacting RNA (piRNA), small nucleolar RNA (snoRNA), and extracellular RNA (exRNA) (35). These classes of short RNAs differ in the biogenesis and their mechanism of action (36). There is another category of non-coding RNA that is the long non-coding RNA (lncRNA). Short non-coding RNAs are directed by protein family, Argonaute (Ago), which guide the RNAs to their specific targets, thereby reducing the expression of the target gene (36). Among the class of non-coding RNA, miRNAs are known to play a pivotal role in the extracellular signaling of the cells. The flow diagram of different small non-coding RNAs is depicted in Figure 1.

Small interfering RNAs (siRNAs) are small (20–25 nt) double-stranded RNAs that act through the RNA interference (RNAi) pathway to interfere with the expression of a specific gene containing a complementary sequence. RNAi can downregulate expression by inducing degradation of the targeted RNA, interfering with transcription or inducing epigenetic changes to the gene (37).

Piwi-interacting RNAs also known as piRNA is a category of small non-coding RNAs with length slightly greater than miRNAs of about 26–31 nt. These categories of RNAs act as gene silencers. piRNA carries an anti-sense sequence to the transposons, thereby targeting the silencing of the transposons (38).

Small cajal body RNA (scaRNA) is an important player in the splicing mechanism, which regulates the spliceosome machinery by biochemically modifying specific nucleotide (39).

The extracellular miRNAs (exRNA) are one of the forms by which non-coding RNAs are exported. These exRNAs are loaded onto high-density lipids (HDLs) or bound by AGO2 protein outside the donor cell, thereby protecting miRNA from RNA degrading enzymes. These are known to regulate cell–cell communication and are used as biomarkers in various diseases as these RNAs circulate stably in the blood stream (40, 41).

Small nucleolar RNA generally range from 60 to 300 nt in length and guide the site-specific modification of nucleotides in target RNAs *via* short regions of base pairing. There are two major classes, the box C/D snoRNAs, which guide 20-*O*-ribose-methylation, and the box H/ACA snoRNAs, which guide pseudouridylation of target RNAs. These RNAs take part in the development of cancer, where they promote transformation, tumorigenesis, and metastasis (42).

Small nuclear RNA (snRNA) is one of the many small RNA species confined to the nucleus; several of the snRNAs are involved in splicing or other RNA processing reactions. The snRNAs are extremely conserved in both primary and secondary structure. Moreover, some residues seem to be completely invariant between organisms. They promote exon ligation and intron excision by acting as a catalyzing agent in the spliceosome machinery and thereby aiding the protein translation (43).

Long non-coding RNAs have received wide attention in gene regulation and are considered as non-protein coding transcripts longer than 200 nt. This nucleotide length limit differentiates long ncRNAs from small regulatory RNAs, such as miRNAs, short interfering RNAs (siRNAs), piRNAs, snoRNAs, and other short RNAs (44).

## Biogenesis of microRNA

First miRNA discovered was lin-4 of 22-nt length and was identified in a gene responsible for postembryonic development in *C. elegans* carrying a complementary sequence in the 3′ UTR of its regulatory target gene (34, 45, 46). There are about 6930 miRNAs discovered in animals and their viruses (47). The miRNA gene is transcribed by RNA polymerase II to produce a primary microRNA (pri-miRNA) precursor molecule whose length varies greatly may be up to 3–4 kb (13, 19, 34, 45, 46). Furthermore, pri-miRNA undergoes nuclear cleavage with sequential actions by nuclear RNase III Drosha and a partner called DGCR8 with double-stranded RNA-binding domain forming a pre-miRNA, 60–70 nt long, as a hairpin structure with two overhangs at their 3′ and 5′ phosphate groups (34, 45, 46). Exportin-5 recognizes the 2-nt overhang generated by RNase III enzyme Drosha at the 3′ end of pre-miRNA hairpin. The protein Exportin-5 (XPO5) plays a crucial role in escorting the pre-miRNA bound to Ran-GTP from the nucleus to the cytoplasm through the nuclear pore (13, 19, 45). Cytoplasmic cleavage of the pre-miRNA occurs with cytoplasmic RNase III Dicer into ~22-nt miRNA duplexes. The Dicer, an endoribonuclease, interacts with the ends and cuts away the loop joining it, generating an imperfect duplex of miRNA:miRNA. In cytoplasm, the Dicer and TAR RNA-binding protein (TRBP) separates the two strands which are incorporated into the RNA-induced silencing complex (RISC), also known as miRISC (45). The strand with higher stability and the stronger base pairing is associated with the silencing complex and other proteins like Argonaute. The weaker strand in aspect of the two criteria can be either degraded or can act as a functional unit, further recruiting the RISC and protein complex (48).

**Figure 2:**
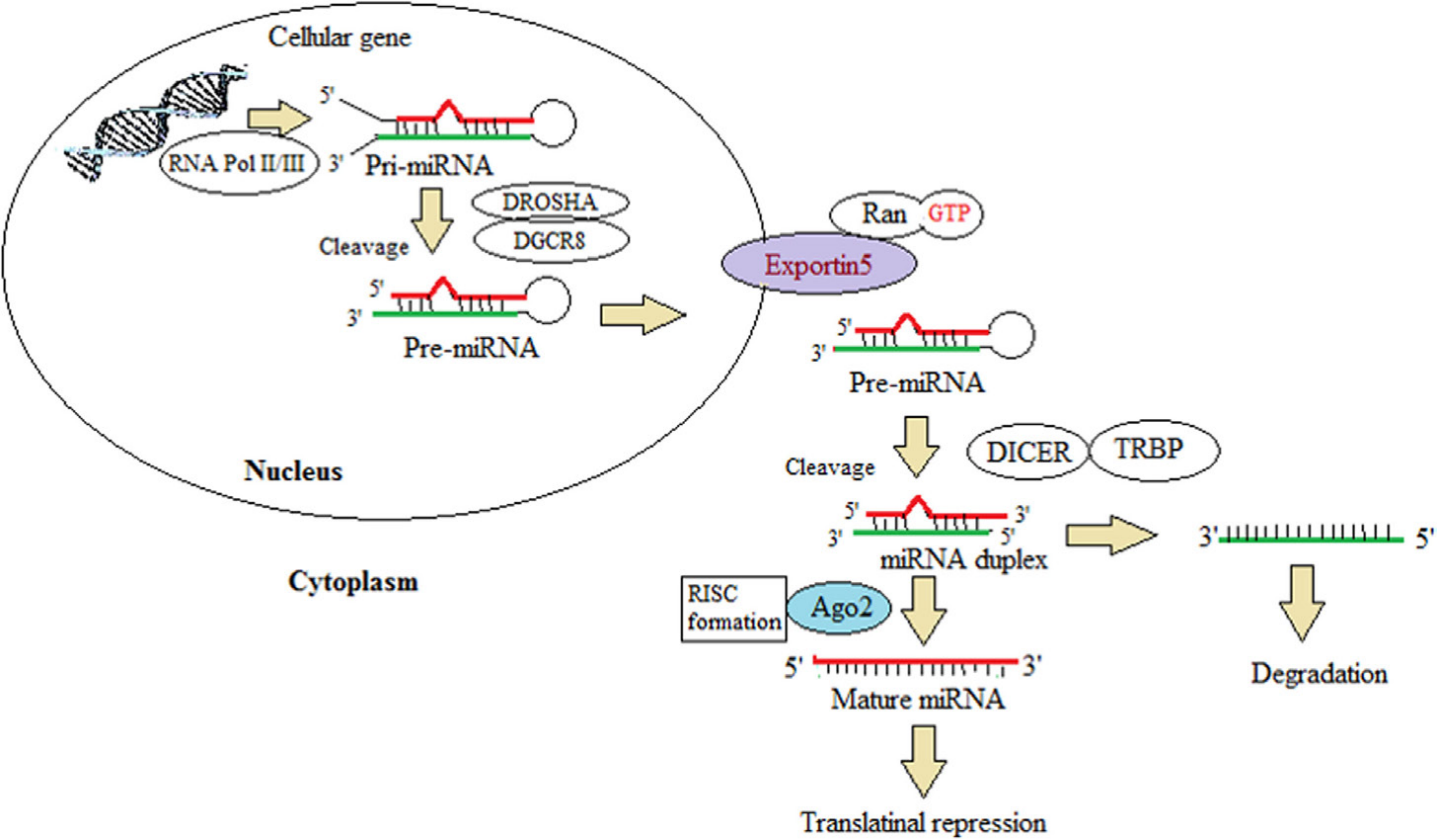
**This illustration depicts the miRNA biogenesis. This represents the canonical maturation where primary miRNA transcripts (pre-miRNAs) are generated by RNA polymerase II or III and cleavage of the pre-miRNAs by the microprocessor complex Drosha–DGCR8 (Pasha) in the nucleus. The pre-miRNAs exit from the nucleus to the cytoplasm by exportin 5 (XPO5)**. In the cytoplasm, it is further processed by DICER1, a ribonuclease III (RIII) enzyme, that gives rise to the mature miRNAs. One strand of the mature miRNA (the guide strand) is loaded into the miRNA-induced silencing complex (miRISC) comprising DICER1 and Argonaute (AGO) proteins. The binding of miRNA to target mRNAs is facilitated by sequence complementary binding, leading to translational repression. This schematic diagram is modified from Lin and Gregory (42).

The outline of miRNA biogenesis is illustrated in Figure 2. miRNA plays an important role in the posttranscriptional regulation by recognizing the specific mRNA and incompletely pairing with the 3′ untranslated region causing translational suppression and inhibition of protein synthesis or by degrading the mRNA. They play a major role in making diverse biological decisions such as proliferation, differentiation, and apoptosis. miRNA can be secreted as circulating miRNA (49) that can either exist outside the cells with the help of various binding proteins or can be exported outside by extracellular vesicles such as exosomes, microvesicles, or apoptotic bodies (50). RNA-binding protein (RBP), Ago-1, and high-density lipopolysaccharides have been reported to assist miRNA out of the cells and prevent its degradation. miRNAs present in the blood plasma are most stable in the microenvironment carrying the property of resisting high and low temperature and varied pH (40). Among all the non-coding RNAs, only miRNAs are seen to play a pivotal role in tumor signaling and also in cardiovascular, neurological, and other developmental diseases (51, 52).

## Packaging and Shipping of miRNAs

**Figure 3:**
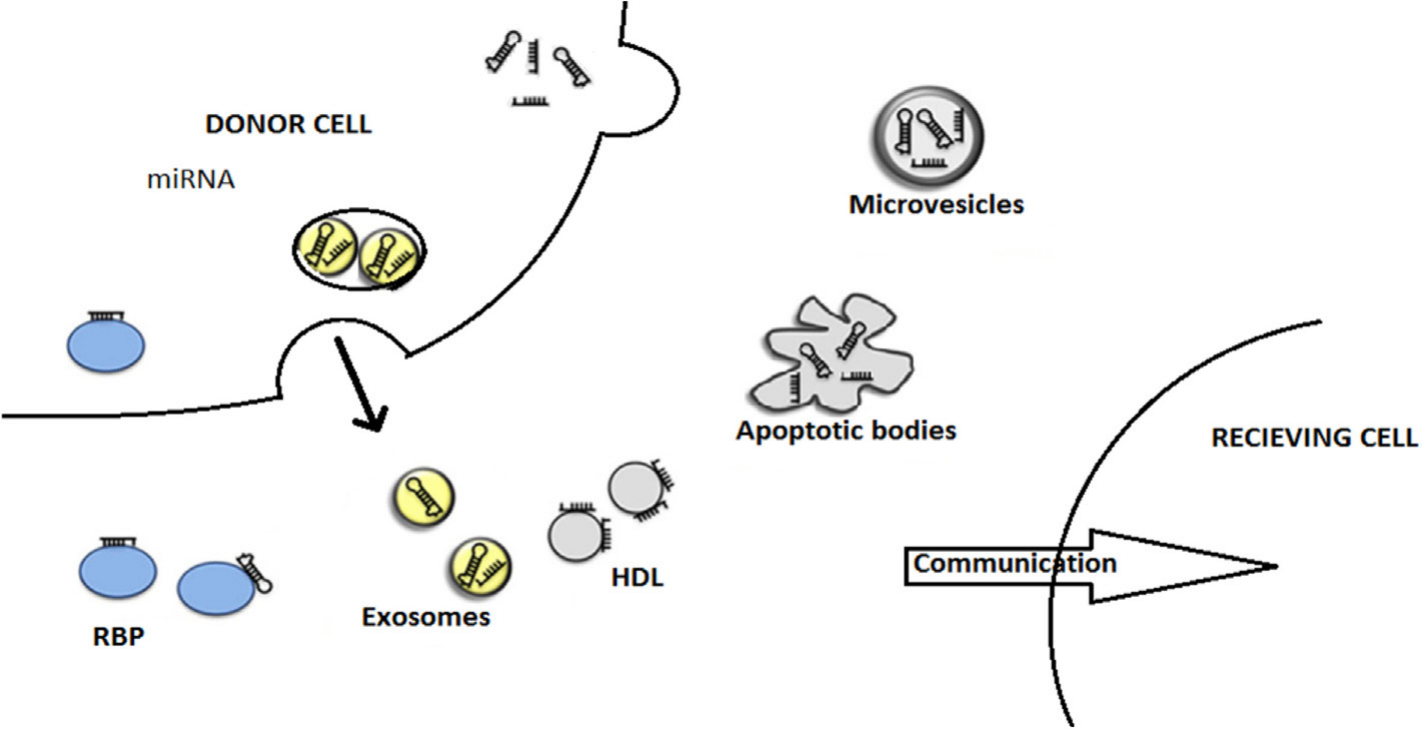
**This figure describes the modes of miRNA export to neighboring cells. The miRNAs are being exported out of the donor cells with the help of various carriers, membrane-derived vesicles (exosomes, microvesicles, apoptotic bodies), miRNA-binding protein complexes (RBPs), or high-density lipoproteins (HDL)**. Finally, miRNAs entered to recipient cells where they are engaged in gene expression alterations. This illustration is modified from Thery (55) and Crescitelli et al. (53).

microRNAs are protected from the potent degrading factors such as RNase during circulation and signaling through the microenvironment. The diagram showing mode of miRNA export is provided in Figure 3. These miRNAs are shielded by two ways, one being encapsulation in the extracellular vesicles, namely, exosomes, microvesicles, or apoptotic bodies (53) and the other being complexing with number of RBPs, such as Ago protein family, or HDL, that occurs in the blood plasma (54). All the three categories of the extracellular vesicles share the same membrane composition, thereby making it tedious to separate out, but they carry different pathways of biogenesis and secretary mechanism (55).

Exosomes are lipid vesicles ranging between 40 and 100 nm in diameters, which arise from the inward budding of plasma membrane of the cell forming an intraluminal vesicle (56). They are associated with the plasma membrane through ligand–receptor interactions (57, 58) or lipids. Exosomes carry a property of taking up various miRNAs, mRNAs, and proteins without eliciting any immune response (59). These vesicles are relatively stable in the cell’s external environment and can easily cross the blood–brain barrier. Exosomes are composed of numerous protein families such as tetraspanin proteins, CD63, CD9, and CD81. Primarily, the cell membrane is internalized to form an endosome, followed by the formation of small vesicles inside the endosomes. These endosomes are then called multivesicular bodies, and they transfer the vesicles into the extracellular space in the form of exosomes (60). The exosome membrane contains lipid rafts containing cholesterol, sphingolipids, glycerophospholipids, and ceramide. The protein content is also significant for the vesicle to travel and target the cell membrane. Various proteins, such as integrin family, cytoskeletal proteins, heat-shock protein family, and vesicle trafficking proteins, play a role in making the vesicular environment habitual for shipping the RNAs and proteins (61).

The levels of signaling *via* exosomes or vesicular transport have seen to be elevated in tumor cells as compared with the normal cells. There are lots of compounds which are studied to act on a target molecule to inhibit the formation of the extracellular vesicles carriers, thereby creating a signaling gap within the tumor microenvironment. Molecules, such as proton pump inhibitors, methyl-β-cyclodextrin, and annexin, are known to inhibit the vesicle formation by targeting the Na^+^ reabsorption, cholesterol, and phosphatidylserine, respectively, and thereby blocking the membrane fusion, lipid raft-mediated endocytosis, and phagocytosis (62).

Microvesicles also referred as shedding microvesicles or ectosomes are small membranous sacs, which are the product of the budding and blebbing of plasma membrane (56, 63). Microvesicles are large vesicles, which are 50–1000 nm in diameter. Microvesicles participate in the cells growth, proliferation, and its death. There are tumor-originated microvesicles, which are secreted into the tumor microenvironment, which helps the neighboring cells to secrete cytokines, which can help the tumor replenish and can mask the immune response (64).

Apoptotic bodies also called apo-bodies arise from the cells undergoing cell death by apoptosis. These are the largest of the extracellular vesicles of 400–4000 nm in diameter. These bodies consist of the condensed chromatin and cytoskeletal proteins (65). As these arise from the shrinkage of the cell, there are many RNA and DNA fragments present whose uptake can result into the transcriptional repression (66).

Extracellular miRNAs are associated with lipid-based carriers and lipid-free proteins (62). Circulating miRNAs are stable when they are associated with plasma, which serves as the disease biomarkers (67). The miRNA-223 is found to be transported using HDL molecule, and the Ago protein family is associated with the extracellular miRNA transport accompanying both in and out of the cell (67). Exosome circulate for a short time in the body fluids before getting internalized into the recipient cell, a process called free floating. Furthermore, adhesion requires a conformational change in the status of the low affinity to high affinity binding to integrin-bound exosome (68). Uptake of exosome is dependent upon the signaling status of the recipient cell and the protein composition of the vesicle (69). Exosome internalization involves various mechanisms such as membrane fusion, soluble signaling, juxtacrine signaling, lipid raft-mediated endocytosis, and macropinocytosis (68). These mechanisms are either receptor-based or involve actin filament protrusions, which engulf the vesicle dispatching the cargo in the cytoplasm (70).

Release of exosomes or microvesicles occurs in the similar fashion like the viral budding (63, 71). The property of the plasma membrane forming a protrusion is driven by a number of proteins and lipids associated with the membrane. The budding of the exosomes *via* endocytosis and membrane trafficking is mediated *via* various coat proteins like clathrins and others like annexing, a Ca^2+^ binding protein, which are present in the cell and which has the ability to recruit phosphatidylinositol 4,5-bisphosphate [PI(4,5)P_2_], sphingolipids, and cholesterol (72). A protein motif called amphipathic helix that acts on the integral site and membrane proteins that bind on the periphery senses the membrane curvatures and are inserted into the membrane, facilitating the budding, and thereby the vesicle formation (69). Lipid rafts play a major role in the promotion of signaling mediated through proteins anchored by glycophosphatidylinositol and help in sorting and membrane trafficking in the secretory pathways (62, 73).

## Communication Bridge between miRNAs to Neighboring Fibroblast

Specific targeted therapies can be achieved by encapsulating the drug into the exosomes with a clear prospect of the therapeutic cargo, the peptide or DNA target of the drug, mode of uptake, and mechanism of their action (74). Some miRNA-targeted therapies are also being designed to deliver antitumor miRNA to the breast tumor cells (75). Exosomes have natural ability to transfer genetic material both locally and systemically, and, therefore, many research groups investigated these vesicles as therapeutic agents. As an instance, microvesicles isolated from endothelial progenitor cells (EPCs) contained proangiogenic miRNA-126 and miRNA-296 (76), and transfer of these miRNAs triggered the activation of the PI3K/Akt signaling pathway and phosphorylation of endothelial nitric oxide synthases and directed endothelial cells to undergo angiogenic and antiapoptotic program (77).

The pivotal role played by the microenvironment-associated resident cells in breast tumor progression has been widely appreciated. In a recent attempt to emphasize the give and take type of exosome-based connection between breast carcinoma and preadipocyte, a type of cellular community in the tumor microenvironment has been reported with clear role of miR-140/SOX2/SOX9 axis that may regulate tumor microenvironment signaling and communication (78). Intentionally diverted glucose metabolisms are a trademark of breast carcinoma. In recent findings, several research groups have attempted to provide evidence that breast carcinoma cells use the secreted vesicle-contained miRNAs as a tool to deprive of the neighboring cells for their food availability, in particular, they target the glucose uptake (79). They have also convincingly mentioned that breast carcinoma may use such strategy to reprogram and progress with destructive metastasis goal (80).

As an evidence of influencing the progression of breast phyllodes tumor xenografts, miRNA-21 enhanced tumor growth and facilitated metastasis by inducing myofibroblast differentiation (81). The recurrence and relapse of breast carcinoma after removal of primary tumor may be driven by well-communicated cell–cell signaling, leading to cancer cell dormancy. In view of the role of secreted miRNAs in maintaining the dormancy of breast cancer cells, evidence has explained that exosomal transfer of miRNA-23b from the bone marrow may promote breast cancer cell dormancy in metastatic settings (82). Such observation will lead to insights for therapeutic approaches by blocking the miRNA-23b-mediated dormancy control in breast cancer cells so that further cell cycling stages of cancer cells will succumb to the drug regimen therapy (82).

The differential expressions of miRNAs in the context of contribution of cancer-associated fibroblast in breast cancer progression are being unraveled. In the same line, the experimental evidence points out that miRNA-26b is highly aberrant miRNAs found in cancer-associated fibroblast and may be implicated in breast carcinoma progression, growth, and recurrence (83).

There is growing view that the cancer-associated fibroblasts and normal fibroblasts being in the neighborhood of breast tumor act differently with the first responsible for inducing breast tumor progression and second engaged in tumor blockade. In this context, about 11 dysregulated miRNAs in cancer-associated fibroblasts have been pinpointed that include three upregulated (miRNA-221-5p, miRNA-31-3p, and miRNA-221-3p) and eight downregulated (miRNA-205, miRNA-200b, miRNA-200c, miRNA-141, miRNA-101, miRNA-342-3p, let-7g, and miRNA-26b) (84). Furthermore, such observation accentuates the notion that miRNAs derived from breast tumor neighboring cells may influence the status of breast tumor through cell–cell communication (85).

The collaboration between stromal cell community and breast tumor cell is being overwhelmingly recognized as a factor behind the tumor progression and development. In recent evidence, there are highlighted reports that suggest that the miRNA-320 is a vital component of the phosphatase and tensin homolog deleted on chromosome 10 (Pten) tumor suppressor axis that works in stromal fibroblasts to manipulate the tumor microenvironment and restrain tumor progression (86). The inducement of senescence has been considered as a blockade for different cancer cell progression, including breast carcinoma. There has been report on the contribution in tumor suppression by senescence-associated microRNAs (SA-miRNAs), miRNA-22, which is overexpressed in human senescent fibroblasts and epithelial cells. Since, such reports validate the idea of communication bridge between stromal cell and tumor cell progression, further studies will provide better understanding and platform for therapy approaches by manipulating the miRNA level in the tumor neighborhood cell community (87).

### The Communication Bridge between Carcinoma and Surrounding Macrophage

In an effort to delineate the collaboration between breast carcinoma and their cellular community in microenvironment, authors have reported that miRNA-21 and miRNA-29a expression are upregulated in tumor-infiltrated myeloid cells and macrophages. Furthermore, authors have suggested that these upregulated miRNAs act as helping hand for the breast tumor for their progression and metastasis (88). In recent time, there are emerging evidence of close association between breast carcinoma and carcinoma-trained or -taught macrophages, in tumor microenvironment. Furthermore, the miRNA profiling data corroborated the observation that deregulated miRNAs expression level is linked with breast tumor progression and prognosis (89). Recent convincing view is that breast tumor-associated macrophages are being extensively causative in the trademark settings of tumor microenvironment. To understand such phenomena, one study suggested that a crucial extra player, miRNA-19a-3p, highly expressed in tumor-associated macrophages may be able to retard breast cancer progression and metastasis through the downregulation of Fra-1 proto-oncogene (90).

The friendly promotion of breast cancer progression and metastasis by tumor-associated macrophages has extensively been highlighted in recent times. In the same direction, there are reports on macrophage-secreted exosome-encapsulating miRNAs such as miRNA-223 that are packed to deliver breast carcinoma and upon entry into the cancer cells resulted into better potentiation of carcinoma cells for their progression and metastasis (91). Recently, appreciation has been received to find the clue behind friendly neighborhood cell community in supporting the survival strategy of breast carcinoma cells.

### Crosstalk between Carcinoma and Stromal Cells

In the line of reported macrophages, fibroblast, and epithelial cells, there are findings on the contribution of human mesenchymal stem/stromal cells (hMSCs) to upkeep breast cancer survival advantages mediated by the secretome. Furthermore, Vallabhaneni et al. (92) have also demonstrated and characterized the role of miRNA-21 and 34a as tumor-friendly miRNAs. Being heterogeneous within the breast tumor milieu, the breast cancer cells are always receptive toward any signals or messages coming from the outside stromal cells populations. To investigate such cell–cell crosstalk, the facts have established that communication between stromal stem cells with the breast carcinoma cells mediated by Twist1 protein player leads to aberrant expression of miRNAs, specifically led by miRNA-199a, which ultimately landed in repression of Forkhead box protein P2 (FOXP2) transcription factor, that may be responsible to encourage breast cancer survival and progression (93).

There has been interest in the clues or signals contributing to molecular alterations in breast tumor stromal environment that actually contribute in the betterment of breast carcinoma. To follow such unrevealed, it was pointed out that upregulation of miRNA-21 in stromal cells may be helping breast tumor for progression and linked it as a prognostic factor (94). The release of extracellular vesicular cargo containing vital messenger in the form of proteins, miRNAs, or tRNAs has been reported from both breast carcinoma and their neighborhood cells, and such cargo gifs are released in the microenvironment to guide or mentor each other type of cells (95). In future, better understanding of molecular interplay involved in the breast tumor microenvironment supporting the breast tumor progression and metastasis may open up the therapeutic intervention strategy or early detection (95, 96).

**Figure 4:**
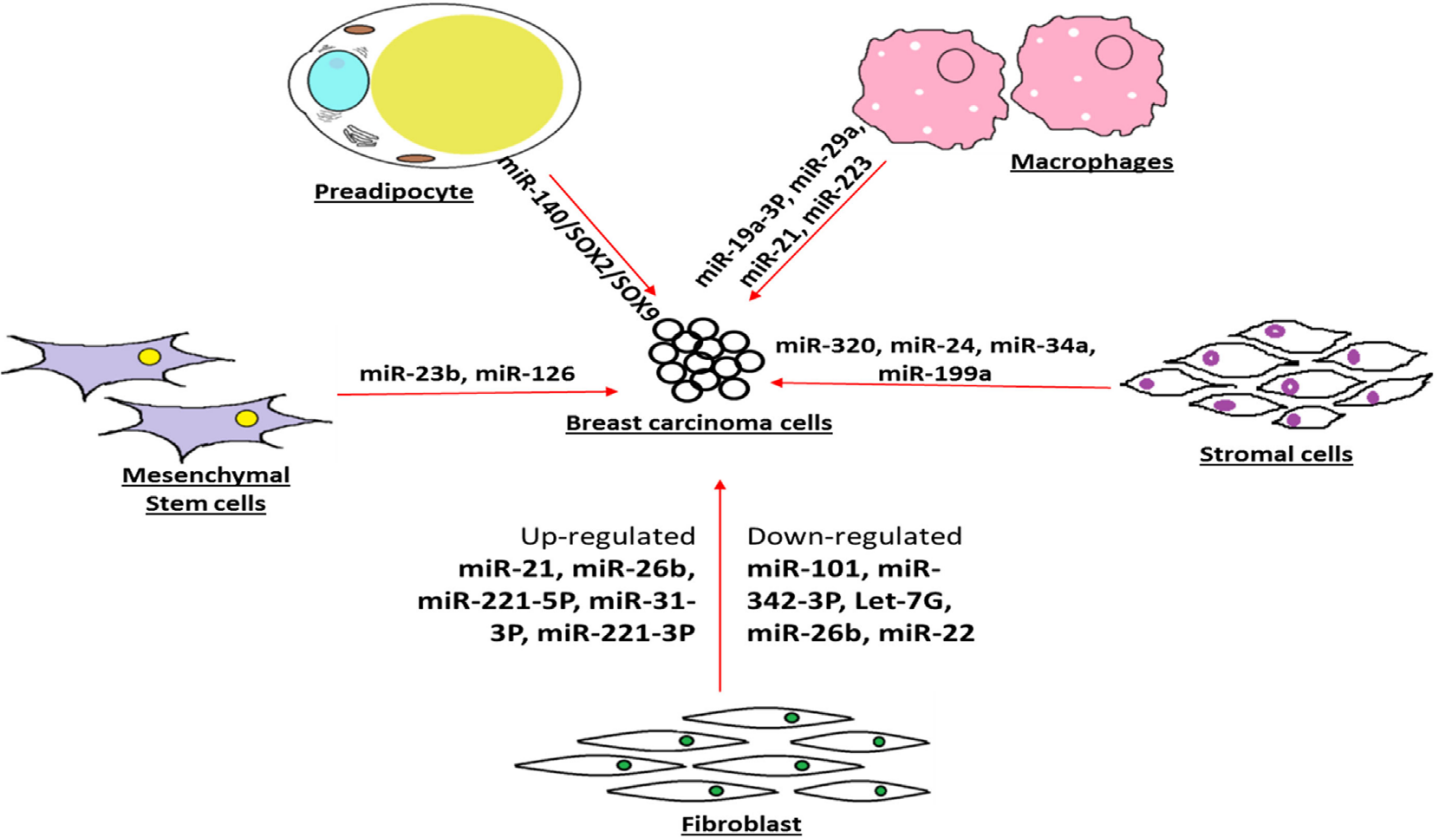
**Enhancement of interaction by miRNAs bridge breast tumor and their microenvironment**.

The contributions of stromal cells in the breast tumor progression are being established through multiple molecular mechanisms, such as augmented invasiveness, angiogenesis, and immunosurveillance masking. The recent experimental data report that miRNA-126/miRNA-126(pair) overwhelms the sequential recruitment of mesenchymal stem cells and inflammatory monocytes into the tumor stroma, leading to the breast tumor metastasis. In other words, downregulation of miRNA-126/miRNA-126(pair) in the breast carcinoma cells may be a strategy to avoid immunosurveillance in tumor (97) and more such examples are depicted in the Figure 4.

### The Summary of Therapeutic Relevant miRNAs

Oncogenic microRNAs (OncomiRNAs) dysregulation causes the onset of cancer. OncomiRs act as negative regulators of the tumor suppressor genes. The most screened miRNAs as prognostic markers of breast cancers are miRNA-10b, miRNA-21, miRNA-373, and miRNA-27a (20, 98). The summarized reviews on oncogenic miRNAs are presented in Table 1.

**Table 1:** **The summary of oncogenic microRNAs reported in literature**.

Oncogenic miRNAs (OncomiRNAs)	Function in breast cancer when overexpressed	Reference
miRNA-10b	Decreases E-cadherin level leading to metastasis Silences tumor suppressor homeoboxD10 (HOXD10) pathway	(99, 100)
miRNA-373	Downregulates CD44 causes migration Makes the cell resistant to apoptosis Promotes cell growth, migration, and invasion	(8, 101, 102)
miRNA-21	Targets PDCD4 and HIF1A gene causing increased cell growth, invasion, and EMT	(103)
miRNA-155	Downregulates FOXO3A gene which causes chemotherapy resistance and reoccurrence of tumor	(104)

### Tumor Suppressor miRNAs

Tumor progression can lead to increase and decrease of some exosome-mediated miRNAs. The decreased miRNAs are known to play a role as suppressor of the tumor. These miRNAs inhibit the growth of tumor by negatively regulating the OncomiRNAs (15, 26, 27). In wide prospects as oncogenic, tumor suppressor, prognosis, and diagnosis tools in cancer, there are appreciable efforts that have been reported in the form of clinical trails, and few examples have been listed in Tables 2 and 3.

**Table 2:** **The summary of tumor suppressor miRNAs reported in literature**.

Tumor suppressor miRNAs	Function in breast cancer	Reference
miRNA 17–92 family	Regulates TGF-β pathway by targeting TGF-β receptor II and the Smad 2 and Smad 4 proteins Targets Mekk2/Mek5/Erk5 pathway enhancing the recognition of NK cells for the tumors	(105, 106)
miRNA 125b	Targets erbB2/erbB3 causing decrease in cell proliferation, differentiation, and induce apoptosis	(107)
miRNA 200	Targets transcriptional repressors ZEB and upregulate the E-cadherin level inhibiting the epithelial to mesenchymal transition (EMT) and cell proliferation	(20, 84)
miRNA-146b	Targets STAT3 and NF-kB signaling pathway	(108, 109)

**Table 3:** **List of miRNAs and their status on clinical trails**.

miRNAs	Details of clinical trails	Reference
microRNA miR-RX34 (MRX34)	A multicenter phase I study of MRX34, microRNA miR-RX34 to evaluate the safety of MRX34 in patients with primary liver cancer or other selected solid tumors or hematologic malignancies	(26, 27, 10)
miRNAs panel	The use of a miRNAs panel to identify thyroid malignancy in FNA leftover cells and the effect of these miRNAs on target genes	(111)
miRNA 200	microRNAs (miRNAs) consisting of 6 miRNA (miRNA-21, miRNA-20a–5p, miRNA-103a–3p, miRNA-106b–5p, miRNA-143–5p, and miRNA-215) was found effective to identify whether one should accept adjuvant chemotherapy or not	(112)
miRNAs panel	Plasma microRNA profiling as first line screening test for lung cancer detection: a prospective study	(113)
miRNAs panel	A perspective study of the predictive value of microRNA in patients with HER2 positive advanced stage breast cancer who were treated with herceptin	(114)
miRNA-29b	The role of microRNA-29b in the oral squamous cell carcinoma	(115)
Circulating miRNAs	Circulating miRNAs: novel breast cancer biomarkers and their use for guiding and monitoring response to chemotherapy	(116)
miRNA-10b	Evaluating the expression levels of microRNA-10b in patients with gliomas	(117)

## Conclusion and Future Aspects

Short RNAs participate in the intracellular as well as intercellular metabolic and developmental signaling pathways in several types of cancer, including breast carcinoma. Decades of research has provided in-depth information regarding the non-coding RNA pathways. miRNAs are known for their ability to communicate with the extracellular microenvironment comprising of cells in its proximity *via* vesicle-derived cargo or conjugated proteins. Levels of various miRNAs differ according to the progression and pathophysiology of breast cancer. Consequently, therapeutic agents such as tumor suppressor miRNA can be designed based on encapsulation in exosome-like vesicle to the neighboring cells of breast tumor for reduced communication. In future, drugs/inhibitors to disrupt friendly communication between breast carcinoma and neighboring cells should be appreciated. Oncogenic miRNAs that are seen to be upregulated in breast tumor and released in their surroundings may used as a biomarkers and diagnostic tools. This review provides the summary in the field of breast cancer pathophysiology focusing on the role of small non-coding RNAs, including miRNA.

## Author Contributions

NS contributed by proposing the concept of the title of review paper, designing the abstract, and drafting the manuscript. DJ and SP contributed by reviewing the literature and converting it into the manuscript form, and drawing of flow diagrams and figures. All authors listed, have made substantial, direct and intellectual contribution to the work, and approved it for publication.

## Conflict of Interest Statement

The authors declare that the research was conducted in the absence of any commercial or financial relationships that could be construed as a potential conflict of interest.
